# Motor Cortical Network Plasticity in Patients With Recurrent Brain Tumors

**DOI:** 10.3389/fnhum.2020.00118

**Published:** 2020-04-03

**Authors:** Lucia Bulubas, Nina Sardesh, Tavish Traut, Anne Findlay, Danielle Mizuiri, Susanne M. Honma, Sandro M. Krieg, Mitchel S. Berger, Srikantan S. Nagarajan, Phiroz E. Tarapore

**Affiliations:** ^1^Biomagnetic Imaging Lab, Department of Radiology and Biomedical Imaging, University of California San Francisco (UCSF), San Francisco, CA, United States; ^2^Department of Neurological Surgery, University of California San Francisco (UCSF), San Francisco, CA, United States; ^3^Department of Neurosurgery and TUM-Neuroimaging Center, Klinikum Rechts der Isar, Technische Universität (TU), Munich, Germany; ^4^Department of Psychiatry and Psychotherapy, University Hospital, Ludwig-Maximilians Universität (LMU), Munich, Germany; ^5^International Max Planck Research School for Translational Psychiatry (IMPRS-TP), Munich, Germany

**Keywords:** brain tumor, magnetoencephalography, motor cortex, neurological surgery, preoperative motor mapping, plasticity, recurrent tumors

## Abstract

**Objective**: The adult brain’s potential for plastic reorganization is an important mechanism for the preservation and restoration of function in patients with primary glial neoplasm. Patients with recurrent brain tumors requiring multiple interventions over time present an opportunity to examine brain reorganization. Magnetoencephalography (MEG) is a noninvasive imaging modality that can be used for motor cortical network mapping which, when performed at regular intervals, offers insight into this process of reorganization. Utilizing MEG-based motor mapping, we sought to characterize the reorganization of motor cortical networks over time in a cohort of 78 patients with recurrent glioma.

**Methods**: MEG-based motor cortical maps were obtained by measuring event-related desynchronization (ERD) in ß-band frequency during unilateral index finger flexion. Each patient presented at our Department at least on two occasions for tumor resection due to tumor recurrence, and MEG-based motor mapping was performed as part of preoperative assessment before each surgical resection. Whole-brain activation patterns from first to second MEG scan (obtained before first and second surgery) were compared. Additionally, we calculated distances of activation peaks, which represent the location of the primary motor cortex (MC), to determine the magnitude of movement in motor eloquent areas between the first and second MEG scan. We also explored which demographic, anatomic, and pathological factors influence these shifts.

**Results**: The whole-brain activation motor maps showed a subtle movement of the primary MC from first to second timepoint, as was confirmed by the determination of motor activation peaks. The shift of ipsilesional MC was directly correlated with a frontal-parietal tumor location (*p* < 0.001), presence of motor deficits (*p* = 0.021), and with a longer period between MEG scans (*p* = 0.048). Also, a disengagement of wide areas in the contralesional (ipsilateral to finger movement) hemisphere at the second time point was observed.

**Conclusions**: MEG imaging is a sensitive method for depicting the plasticity of the motor cortical network. Although the location of the primary MC undergoes only subtle changes, appreciable shifts can occur in the setting of a stronger and longer impairment of the tumor on the MC. The ipsilateral hemisphere may serve as a reservoir for functional recovery.

## Introduction

The human brain contains eloquent regions of particular significance to specific neurological functions. A lesion, such as a tumor, within these eloquent areas, is likely to give rise to neurological deficit. Although eloquent regions are present in every brain, their exact locations are variable and must be defined on a case-by-case basis. Moreover, recent studies have demonstrated that areas of eloquence can move over time, in a process of reorganization known as cortical plasticity (Duffau, 2001; Tecchio et al., 2006; Robles et al., 2008; Southwell et al., 2016). Functional reorganization likely takes place via several mechanisms, many of which are still under investigation. One such mechanism involves the recruitment of compensatory areas, specifically the ipsilateral non-primary, or the contralateral primary and non-primary motor cortices (Weiller et al., 1993; Seitz et al., 1995; Duffau et al., 2002; Bulubas et al., 2016). Functional reorganization is particularly important in patients with slow-growing brain tumors, in whom the competing pressures of tumor progression and cortical adaptation create an evolving spatial relationship between lesion and surrounding cortex. For such a patient, functional reorganization may dictate whether a lesion is operable and, if not, whether it may be so in the future, thus having profound implications on length and quality of life.

Magnetoencephalography (MEG) is a modality that can accurately and noninvasively identify regions of motor eloquence (Taniguchi et al., 2004; Tecchio et al., 2006; Willemse et al., 2010). In patients with brain tumors, MEG is a consistent, sensitive and specific method for preoperative localization of motor function (Nagarajan et al., 2008; Tarapore et al., 2012). In brief, the process works as follows: during a motor task, the activated motor cortical network changes the β-band frequency, a phenomenon known as event-related desynchronization (ERD). MEG locates the source of these ERDs, thereby identifying the cortical region associated with the motor task (Pfurtscheller and Lopes da Silva, 1999; Pfurtscheller, 2001). Two main approaches are used for source location: the dipole fitting method (Salmelin and Hämäläinen, 1995), and the synthetic aperture magnetometry (SAM) beamforming approach (Vrba and Robinson, 2001; Nagarajan et al., 2008). MEG-based motor mapping gives results that are consistent with functional magnetic resonance imaging (fMRI), navigated transcranial magnetic stimulation (nTMS) and direct cortical stimulation (DCS; Ganslandt et al., 1999; Schiffbauer et al., 2002; Castillo et al., 2004; Nagarajan et al., 2008; Tarapore et al., 2012). MEG-based motor mapping can be performed at regular intervals and used to track the movement of the motor system. It is, therefore, an ideal modality for characterizing cortical reorganization over time.

In this investigation, our primary objective was to characterize the reorganization that takes place in the motor system of patients with progressing brain tumors. From a population of patients that presented at our Department for tumor resection surgery, we identified the cohort who had recurrent tumors and underwent tumor resection and MEG-based motor mapping as part of preoperative assessment at two or more time points. We used these maps to identify changes in the location of the motor cortical network and to characterize differences in its pattern of activation. Our secondary objective was to identify any factors influencing that reorganization. We subsequently examined demographic, pathological, and clinical variables to identify factors that independently influence these changes. In so doing, we demonstrate that both contralateral and ipsilateral motor cortices are capable of functional reorganization in patients with primary brain tumors, and that greater time between scans and presence of a motor deficit are associated with greater cortical reorganization.

## Materials and Methods

### Study Design

We retrospectively analyzed preoperative MEG data of 78 consecutive patients with primary brain tumors who were scheduled for surgical brain tumor resection between 2003 and 2016 at our department. At our University, functional imaging based on MEG for locating motor and speech function is part of the pre-operative routine. This study retrospectively investigated these routinely collected data in patients who presented for at least two tumor resection surgeries, i.e., patients who got a recurrent tumor after their first tumor resection surgery. This study’s experimental protocol was approved by the Institutional Review Board at UCSF, and all research was conducted according to approved protocols consistent with the Declaration of Helsinki. Written informed consent is collected prospectively from all patients undergoing MEG motor mapping and was available for all subjects in this study.

### Patient Population

The patient population was compiled from an initial list of 99 patients with primary brain tumors, known to have received multiple MEG scans at the Biomagnetic Imaging Laboratory. MEG scans of motor activity were collected from every patient as part of routine pre-operative functional imaging typically conducted on the day before surgery. Exclusion criteria were low-quality or missing MEG-based motor mapping (10 patients) and bilateral tumors (three patients). Inclusion criteria were a glial neoplasm, and a minimum of two MEG motor mapping scans. For the activation peak analysis, an additional exclusion criterion was the inability to localize the primary motor cortex (MC) using the adaptive spatial filtering approach (11 patients). From these 11 motor mapping scans, three were eligible for the second analytical approach, the spatial analysis of whole-brain maps, resulting in a total of 78 patients included in at least one of the analyses (see Supplementary Material).

Patient data were extracted from our university’s electronic medical record system. This patient information provided insight into relevant variables (sex, tumor side, handedness, the dominance of tumor hemisphere, tumor location, tumor entity, patient’s motor deficit, and status post radiation or chemotherapy) and allowed further grouping of the patients by these markers (Table 1). A board-certified, licensed neurosurgeon (PT) revalidated tumor side and location from the original magnetic resonance imaging (MRI) scans on a radiology workstation. Tumor size, location, and pathology were defined based on the timing of the first MEG scan. We assessed whether the tumor extended each into the frontal, parietal, temporal, or insular lobe. Handedness was determined according to patient self-reporting. Motor-dominant hemisphere was defined as the left hemisphere in right-handed and the right hemisphere in left-handed patients. Two ambidextrous patients were excluded from the dominance analysis. Motor deficit was defined using the British Medical Research Council (BMRC) scale as muscle strength <5/5 in at least one of upper or lower extremity muscles during preoperative full body examination; motor deficits, history of radiation therapy, and history of chemotherapy were determined based on the timing of any MEG scan.

**Table 1:** Patient characteristics.

Total number of patients		*n* = 78
Sex	Female	34 (44%)
	Male	44 (56%)
Tumor side	Left	46 (59%)
	Right	32 (41%)
Handedness	Left	10 (13%)
	Right	66 (85%)
Dominance of tumor hemisphere	Dominant	43 (55%)
	Non-dominant	33 (43%)
Tumor located predominantly in	Frontal, parietal, and frontal-parietal lobes	29 (37%)
	Frontal-insular-temporal and temporal lobes	49 (63%)
Entity	WHO grade II glioma	44 (56%)
	WHO grade III glioma	20 (26%)
	WHO grade IV glioma	14 (18%)
Motor deficit	Full strength	66 (85%)
	Motor deficit	12 (15%)
Radiation therapy	No history of	53 (68%)
	History of	25 (32%)
Chemotherapy	No history of	19 (24%)
	History of	59 (76%)
Patient age at	First timepoint	43.7 ± 12.0 (18, 74 years)
	Second timepoint	46.6 ± 11.6 (22, 75 years)
	Third timepoint (*n* = 11)	49.8 ± 6.3 (40, 62 years)
Days passed	First to second timepoint	1080 ± 800d (12.6 years; 63, 4603d)
	First to third timepoint (*n* = 11)	717 ± 326d (3.7 years; 336, 1345d)

*This table provides details on the characteristics of a total of 78 enrolled patients. Abbreviations: n, number of patients*.

### Magnetic Resonance Imaging (MRI)

At the time of each MEG session, patients also underwent a high-resolution structural MRI scan on either a 1.5 or 3 Tesla scanner. Series for 1.5 Tesla scans typically included the following: 1) a T1-weighed, 3D spoiled gradient-recalled echo (spgr) sequence with a 34 ms TR, 3–6 ms TE, and 35° flip angle, and 2) a T2-weighted 3D fast spin-echo (fse) sequence with a 2.6–4.0 s TR, 104 ms TE, and 90° flip angle. Both sequences had a slice thickness of 1.5 mm, a 256 × 192 acquisition matrix, and contained between 110–132 slices. Series for 3 Tesla scans typically included the following: 1) an spgr sequence with a 6–9 ms TR, 2–3 ms TE, and 12–15° flip angle, and 2) a fse sequence with a 2.0–3.8 sTR, 87–159 msec TE, and 90° flip angle. Both sequences had a slice thickness between 1–1.5 mm, and the acquisition matrix from 256 × 256 to 288 × 288, contained between 114–428 slices, and included all fiducial points and markers. At each time point, the MEG scans were coregistered with the structural scans obtained within a few days of the MEG scan based on anatomical landmarks, such as the nasion and the left and right auricular points. Our analysis pipeline excludes any data with coregistration errors exceeding 0.5 cm for each session. The same markers were used for the coregistration of the MRI and MEG throughout the sessions and the markers were placed by the same experienced technicians (AF, DM, SH), ensuring a consistent placement.

### Magnetic Source Imaging

Magnetic fields were continuously recorded in a shielded room using a 275-channel whole-head CTF Omega 2,000 system (CTF Systems, Inc., Coquitlam, BC, Canada) while the participants were lying awake, with their eyes closed, or during a task. The MEG signals were digitized at a sampling rate of 1,200 Hz. MEG scans were performed in resting state and during somatosensory, motor, and language tasks to locate respective brain function. For detection of the motor cortical network, the patients performed a specific motor task which consisted of self-paced unilateral index finger button press once every 2.5–4 s for a total of 100–250 movements, as described earlier (Nagarajan et al., 2008; Tarapore et al., 2012). The motor task was performed for left and right index fingers separately.

The MEG data obtained during the motor task was bandpass-filtered in the β-band. Usually, the 15–30 Hz frequency was used. For motor peak analysis, if no peak activation was seen in the primary MC at this frequency, and 30–50 Hz (or, for some patient, 30–55 Hz) better resembled a motor activation and gave a peak activation in the MC, this frequency was exceptionally used. To correct for sensory activation, sensor data covariance was computed in a window beginning approximately 600 ms before the onset of movement (marked by the button press, i.e., the “active period”) as well as for a 600 ms baseline period timed while there is no movement, 0.9–1.5 s after the completion of movement (also marked by button press, i.e., the “control period”). Artifact rejection was performed by: (1) exclusion of bad trials that exceeded 1 pT fluctuations established using an automated procedure; and (2) restriction of head movement to within 0.5 cm during a scan, and eventually repeating scans if they exceed this movement threshold. To analyze the β-band ERD, an adaptive spatial filtering algorithm was used, as described in the literature (Sekihara et al., 2001, 2004; Vrba and Robinson, 2001). In short, an estimate of the source power at each voxel in the brain based on the MEG data was computed for the active and control periods. This was performed using a forward field, which was computed assuming a multiple local-sphere spherical volume conductor model and making use of the sensor data covariance. The resolution for source power estimates across the entire brain was set at 5 mm for the active and control periods. A pseudo-F ratio was calculated, in which negative values indicate ERD and positive values indicate event-related synchronization (ERS). ERD represents an increase in neural activation during motor tasks, which indicates the motor function, and ERS represents a decrease in neural activation. This whole-brain ERD/ERS images represent the results of the preoperative motor mapping, the individual motor maps. Adaptive spatial filtering was performed using the SAM software package (CTF Systems, Inc., Coquitlam, BC, Canada) and integrated using the NUTMEG software suite^1^ (Dalal et al., 2004), which runs under MATLAB (The MathWorks, Inc., Natick, MA, USA) in conjunction with SPM8^2^.

### Spatial Analysis of Whole-Brain Motor Maps

Tumor location affects motor pathways, yet it is not possible to predict in which direction or to which extent. To minimize this variance, we wanted to perform the analysis of whole-brain spatial motor maps in groups of patients with very similar tumor location. We classified the tumor location in terms of its extent to the frontal, parietal, temporal, and insular lobes. One third (*n* = 27) of our sample had a tumor that extended into the frontal and temporal lobes, as well as the insula, while other tumor location groups had less than 20 patients, a number not likely sufficient for imaging analyses. Hence, the whole-brain spatial analysis was limited to the largest subgroup of patients with frontal-insular-temporal tumors (*n* = 27). We used the β-band ERD/ERS images to analyze the changes in activation patterns of the whole brain. For each session (time point 1 and time point 2), the motor maps were first coregistered with the corresponding structural MRI scan as described above and the MRI scan and the motor maps both were normalized to standardized Montreal Neurologic Institute template space. Spatial normalization was performed using the SPM software^3^) and statistical analyses using the NUTMEG MEG analysis toolbox^4^ (Dalal et al., 2004). The normalization results were manually checked for congruence of anatomic landmarks such as the skull, ventricles, and the precentral gyrus. Spatial normalization threshold errors were within 0.5 cm which is at the spatial resolution of MEG reconstructions of motor cortical activity.

The normalized whole-brain motor maps from the first and the second time point were then tested for a difference in activation of motor pathways using a paired *t*-test, specifically by calculating the difference of condition 1 (ERD/ERS images at first timepoint) minus condition 2 (ERD/ERS images at second timepoint). To focus our analysis on cortical network reorganization, a cortical mask was applied for this statistical comparison. The two time-points were compared using nonparametric randomization statistical tests. To reduce false positives due to multiple comparisons cluster corrections were applied (Eklund et al., 2016). For each tumor subgroup analysis, our criterion was *p* < 0.05 with a cluster size threshold of 20. When combining the different tumor subgroups (as in Figure 3), we applied a more stringent threshold of *p* < 0.01, with a cluster size threshold of 20. First, we performed the analysis separately with motor maps of the left and right index finger and subgroups of left-sided and right-sided tumors. We then investigated how primary MC may be affected by a tumor in the same hemisphere (i.e., how is right-sided primary MC affected by a right-sided tumor and *vice versa*). This analysis was achieved by flipping the normalized left index finger motor maps of patients with right-sided tumors along the x-axis (thereby inverting their laterality), aggregating them with the normalized right index finger motor maps of left-sided tumors, and using unpaired *t*-test across timepoint 1 and time point 2.

**Figure 1:**
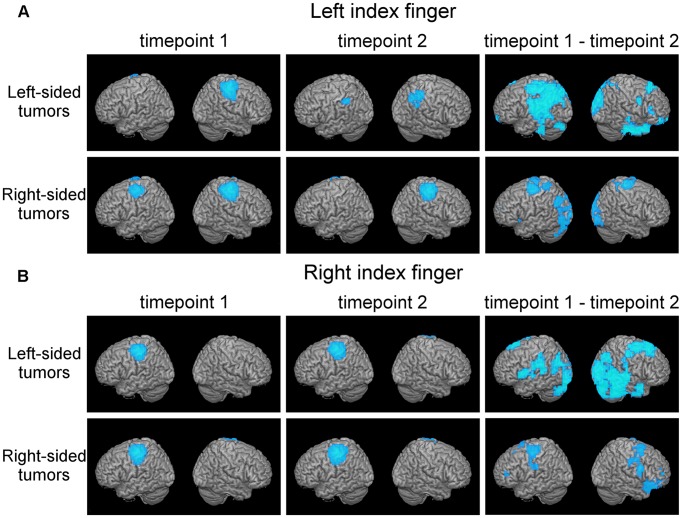
Plasticity in motor cortical activation patterns across observational periods reveals shifts in the contralateral motor cortex (MC) and disengagement of ipsilateral motor cortical regions. This figure shows average motor activation (thresholded at full-width-at-half-maximum) at first (left columns) and second (middle column) timepoint, respectively, in patients with frontal-insular-temporal tumors. Also, statistically significant changes in activation patterns (first minus second time point; blue areas indicate decreased activation at second timepoint) are shown (right column, *p* < 0.05 with a cluster threshold of 20 voxels). Rows indicate different tumor locations. Activation during both the left index finger **(A)** and right index finger **(B)** motor task is shown.

**Figure 2:**
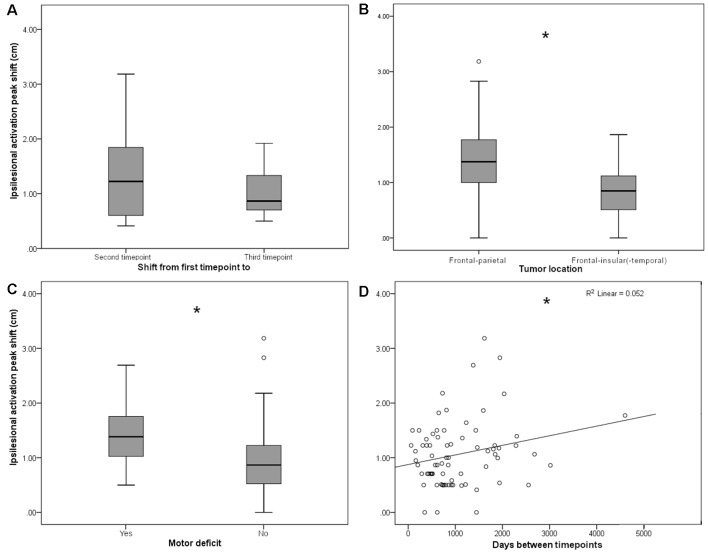
**(A)** Persistent shifts are observed with longer follow-up times. Box plot shows Euclidian distance shift from first to second and from first to third time point, respectively, in the subgroup (*n* = 11) of patients with observations from three time points. No significant difference was observed. **(B)** Ipsilesional MC peak shifts are greater in perirolandic regions. Box plot shows Euclidean distance-based shifts in the activation peak in tumors exceeding into the frontal and/or parietal lobes, as opposed to tumors exceeding predominantly into frontal-insular and/or temporal lobes. Patients with frontal/parietal tumors showed largest primary MC shifts (*t*_(73)_ = 4.38; *p* < 0.001). **(C)** Ipsilesional MC peak shifts are greater for patients with motor deficits. Box plot shows Euclidian distance shifts in the activation peak for patients with or without motor deficits. Patients with motor deficits showed larger primary MC shifts (*t*_(73)_ = −2.36; *p* = 0.021). **(D)** Shifts show linear time-dependence between scans. Effect of days between the first and the second time point on the activation peak shift. Days between timepoints accounted for 5.2% of the variance in activation peak shift with a regression equation of 0.88 + 0.18*10^−3^, showing a direct correlation of days between timepoints and ipsilesional MC shifts (*F*_(1,73)_ = 4.04, *p* = 0.048; fitted regression line *R*^2^ = 0.052). *p < 0.05.

**Figure 3:**
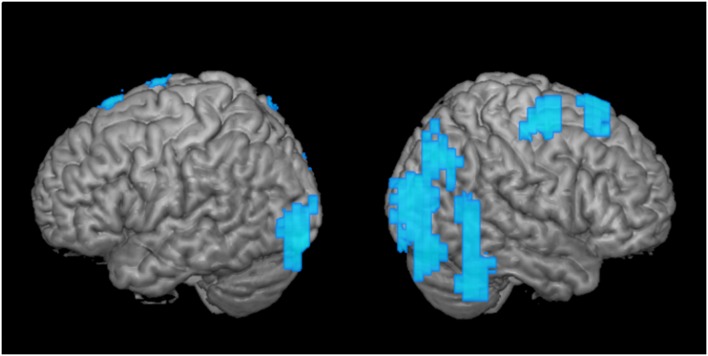
Greater disengagement observed in the contralesional hemisphere. Statistically significant changes in activation maps from first to the second time point when the tumor was located in the proximity of the primary MC (shown left; displayed are left-sided tumors with right index finger activation maps, and right-sided tumors with left index finger activation maps that were flipped along the x-axis). Blue areas indicate decreased activation at the second time point of the ipsi- (left) and contralesional (right) activation maps in patients with frontal-insular-temporal tumors. The level of significance was set at *p* < 0.01; results were corrected at a cluster level of 20 voxels.

### Use of Activation Peaks to Locate the Primary MC

Seventy-five patients where one activation peak in the primary MC could be determined were considered for this analysis. The β-band ERD/ERS images in subject-space were used to calculate activation peaks of ERD using the built-in SAM beamformer algorithm from the CTF software (CTF Systems, Inc., Coquitlam, BC, Canada) with a minimum separation of 10 mm between the peaks. Experienced MEG technologists (AF, DM, SH) chose the strongest peak located in the precentral gyrus of the hemisphere contralateral to finger movement as the representation of the primary MC.

For this analysis, we exported in head coordinates (x, y, and z in cm) for each patient, their activation peak of the left and right MC in subject-space (Tarapore et al., 2012).

Distances between activation peaks were calculated based on Euclidian distance:

$$\sqrt{{\left(\text{x}\left[\text{timepoint(tp)2}\right]-\text{x[tp1]}\right)}^{2}+{\left(\text{y[tp2]}-\text{y[tp1]}\right)}^{2}+{\left(\text{z[tp2]}-\text{z[tp1]}\right)}^{2}}$$

A paired *t*-test was used to determine differences between the activation peak shifts. All results are presented as mean values ± standard deviation. To display the relocation in different spatial axes, we calculated the absolute shifts of single activation peak coordinates between first and second scans (*n* = 75) and first and third scan (*n* = 11), respectively. Also, we calculated the interclass correlation coefficient (ICC) of average measurements for each x-, y-, and z-coordinate using a 2-way mixed effect model (McGraw and Wong, 1996). The ICC scores are customarily used to determine test-retest reliability; ICC = 1 indicates perfect reliability, ICC > 0.80 shows almost perfect reliability, 0.61–0.80 substantial reliability, 0.41–0.60 moderate reliability (Landis and Koch, 1977). We used the ICC to determine coordinates with lowest ICC scores indicating the axis most prone to movement.

Additionally, nine independent variables (age, days between scans, sex, dominance, tumor location, entity, motor deficit, radiation, and chemotherapy) were tested for influence on ipsi- and contralesional activation peak shifts. To test differences between groups, *t*-test (p parametric = p_p_) and Mann Whitney U test (p nonparametric = p_np_) were used. Shapiro Wilk test was used to assess normality distributions and showed a non-normal distribution of contralateral MC activation peak shifts. Univariate linear regression analysis was performed to test the effects of continuous variables. The standardized residuals of the linear regression were plotted against the standardized predicted values, suggesting a normal distribution of the residuals. Additionally, multiple regression analysis was performed, including factors that showed statistically significant effects in the univariate analysis. For all statistical tests, the level of significance was set at *p* < 0.05. All statistical analysis was performed using SPSS (IBM SPSS Statistics for Windows 20.0, Armonk, NY, USA).

## Results

### Clinical Overview of Patient Sample

Patients were predominantly male (44 males, 34 females), right-handed (66 right-handed, 10 left-handed), and had a left-sided (46 left, 32 right) WHO grade II (WHO grade II 44, WHO grade III 20, WHO grade IV 14) glioma. Thirty-eight patients were newly diagnosed at the first time point of our study with no previous treatment, while 40 underwent tumor resections in the past. At first timepoint, five patients have had a motor deficit, four presented with sensory deficit, and two with speech deficits. All patients underwent a tumor resection surgery, usually the day after first MRI and the first MEG scan were performed, and the standard post-treatment according to the surgery outcomes. At the second time point, nine patients presented with a motor deficit, seven of these patients other than those with motor deficits at first time point; four had sensory deficits, and 10 had speech deficits. In those patients who visited on a third time point, there were no motor deficits, three had sensory and two speech deficits. Most patients have originally presented with a history of one or more seizures; in five cases, the tumor was asymptomatic, seven patients suffered from headaches only, two had predominantly motor symptoms. Four patients suffered from psychiatric symptoms, such as personality change and depression. Only for three patients, cognitive deficits such as memory loss were recorded in our medical system, but there was no cognitive testing performed.

### Plasticity in Whole-Brain Activation Patterns

Discrete shifts of the primary MC were observed in whole-brain activation patterns from first to second time point in the 27 patients with frontal-insular-temporal tumors (first two columns, Figures 1A,B). For both left and right finger movement maps the group effects were driven mainly by the left-sided tumors. In this group of left-sided tumors, a strong disengagement of motor activity in the hemisphere ipsilateral to the movement (in particular the left hemisphere during left finger movement) was seen at the second timepoint. These shifts were statistically significant as represented by generally decreased activation (blue areas), specifically in Brodmann areas (BA) 3, 4, and 6 (third column, upper row, Figure 1A).

### Shifts in Activation Peaks

On average, both ipsi- and contralesional activation peaks moved considerably in all 75 patients from first to second scan (ipsilesional 1.06 ± 0.60 cm, contralesional 0.98 ± 0.50 cm), with no significant difference between ipsi- and contralesional shifts (*p* > 0.05). From first to the third scan, the ipsilesional activation peaks moved in all 11 patients with 3 MEG scans 1.04 ± 0.43 cm and the contralesional activation peaks moved 0.89 ± 0.42 cm; they did not move significantly more than from time point 1 to time point 2 (*p* > 0.05, Figure 2A).

Calculations of single coordinate shifts and ICC scores indicated a relative stability of activation peaks compared to the whole-brain activation, shown by rather high ICC scores (Table 2). Greatest ICC scores were found for the x-coordinate, demonstrating little movement along the posterior-anterior axis, followed by the z coordinate, demonstrating movement along the inferior-superior axis, and major movement was found along the left-right axis, as shown by the y coordinate (Table 2).

**Table 2:** Shift of single activation peak coordinates and the respective interclass correlation coefficient.

	Coordinates	Coordinate shift		Reliability	
		Mean ± SD	(min; max)	ICC	Sig.
**From first to second time point, *n* = 75**					
Left index finger	x	0.56 ± 0.46	(0.00; 2.00)	0.78	<0.0010
	y	0.52 ± 0.42	(0.00; 1.70)	0.57	<0.0010
	z	0.51 ± 0.44	(0.00; 2.50)	0.69	<0.0010
Right index finger	x	0.51 ± 0.45	(0.00; 2.00)	0.83	<0.0010
	y	0.54 ± 0.47	(0.00; 2.10)	0.48	<0.0029
	z	0.38 ± 0.39	(0.00; 1.50)	0.79	<0.0010
**From first to third time point, *n* = 11**					
Left index finger	x	0.39 ± 0.28	(0.00; 0.80)	0.82	<0.0010
	y	0.49 ± 0.31	(0.00; 1.00)	0.69	<0.014
	z	0.60 ± 0.33	(0.00; 1.00)	0.45	<0.081
Right index finger	x	0.61 ± 0.45	(0.00; 1.50)	0.89	<0.0010
	y	0.49 ± 0.40	(0.00; 1.30)	0.61	<0.042
	z	0.32 ± 0.37	(0.00; 1.00)	0.92	<0.0010

*This table reports the relocation of activation peak coordinates from first to second MEG scan for the 75 patients with activation peaks within the motor cortex; 75 patients presented for two MEG-based motor mappings, and 11 patients from the first group presented for a thirs MEG-based motor mapping subsequently. We report the respective interclass correlation coefficient scores for motor peak head coordinates. Abbreviations: SD, standard deviation; min, minimum value; max, maximum value; ICC, interclass correlation coefficient; Sig., significance value; n, number of patients*.

### Factors Influencing the Shifts in Activation Peaks

Although the overall activation peak shifts were rather small, we were still able to determine factors that accounted for these subtle shifts of the primary MC. Tumor location, presence of motor deficit, and days between scans showed an effect on the movement of ipsilesional activation peaks, whereas no effect was seen for other factors (Table 1) or contralesional activation peaks. Patients with tumors exceeding into the frontal and/or parietal lobes (as opposed to tumors exceeding predominantly into frontal-insular and/or temporal lobes, *t*_(73)_ = 4.38; p_p_ and p_np_ < 0.001, Table 3, Figure 2B), motor deficits (*t*_(73)_ = −2.36; p_p_ = 0.021 and p_np_ = 0.015, Table 4, Figure 2C), and patients with a longer period between scans (*F*_(1,73)_ = 4.04, *p* = 0.048, Figure 2D) showed larger activation peak shifts.

**Table 3:** Activation peak shifts in tumor location subgroups.

Tumor location	*n*	Ipsilesional hemisphere	Healthy hemisphere
		Mean ± SD	Mean ± SD
Frontal, parietal, and frontal-parietal lobes	29	1.41 ± 0.73	1.10 ± 0.54
Frontal-insular-temporal and temporal lobes	46	0.84 ± 0.38	0.90 ± 0.47

*This table reports the activation peak shifts from first to second MEG scan, calculated using the Euclidian equation, in subgroups of patients according to their tumor location. Abbreviations: SD, standard deviation; n, number of patients*.

**Table 4:** Activation peak shifts in motor deficit subgroups.

Patients with	*n*	Ipsilesional hemisphere	Healthy hemisphere
		Mean ± SD	Mean ± SD
No motor impairment	63	0.99 ± 0.58	0.99 ± 0.50
Motor deficit	12	1.43 ± 0.62	0.91 ± 0.54

*This table reports the activation peak shifts from first to second MEG scan, calculated using the Euclidian equation, in subgroups of patients according to their motor deficit. Abbreviations: SD, standard deviation; n, number of patients*.

Additionally, by using a multiple linear regression model, we were able to predict the ipsilesional activation peak shift based on the motor deficit, tumor location, and days between scans. The regression model accounted for 22.6% of the variance in MC shift (*F*_(3,74)_ = 8.21, *p* < 0.001). Only tumor location added statistically significantly to the prediction [p_p_ (tumor location) < 0.001 and p_np_ = 0.01].

The same analysis was performed in patients for whom scans from three time points were available (*n* = 11). In this analysis, we examined the same independent variables for their effect on the activation peak shift between the first and the third time points. Only tumor location affected the ipsilesional activation peak shift, and the largest shifts occurred in patients with tumors located in frontal and/or parietal lobes (*t*_(9)_ = 4.45, *p* = 0.0016; Table 5).

**Table 5:** Ipsilesional activation peak shifts from first to third scan in tumor location subgroups.

Tumor location	*n*	Ipsilesional hemisphere
		Mean ± SD
Frontal, parietal, and frontal-parietal lobes	6	1.36 ± 0.34
Frontal-insular-temporal and temporal lobes	5	0.65 ± 0.10

*This table reports the ipsilesional activation peak shifts from first to third scan, calculated using the Euclidian equation, in subgroups of patients according to their tumor location. Abbreviations: SD, standard deviation; n, number of patients*.

### Ipsilesional and Contralesional Whole-Brain Motor Activations

As a second step, we analyzed the effect over time of tumors in the same hemisphere as the primary MC upon motor cortical network activation maps (Figure 3; tumor is represented in the left hemisphere). When we compared these activation maps at the first and the second time points, we observed a clear disengagement of motor activity in the contralesional hemisphere (i.e., ipsilateral to finger movement). These changes manifested mainly in BA 4 and 6, as well as posterior regions (Figure 3). Interestingly, no change was observed in the ipsilesional, primary MC itself.

## Discussion

We observe plasticity in motor cortical activation patterns across observational periods. Plasticity is manifested both as shifts in the ipsilesional motor cortical network and disengagement of the contralesional motor cortical network. Ipsilesional MC peak shifts are greater in tumors located predominantly in frontal-parietal regions and for patients with motor deficits, both indicating a stronger impairment of the tumor on the MC. Persistent shifts were observed with longer observational periods. These findings suggest that there are several mechanisms involved in the plasticity of motor cortical areas in patients suffering from gliomas. Understanding these mechanisms is critical to the safe and effective management of these lesions.

These investigations are based on a large, retrospectively analyzed cohort of patients who underwent at least two tumor resection surgeries, each preceded by a MEG-based motor mapping. Several surgical interventions and naturalistically determined observational periods in our sample make disentangling the effects of plastic reorganization difficult, yet, to our knowledge, this is the largest cohort of multi-staged, MEG-based motor maps to date. To maximize the homogeneity of our sample, we limited our examinations to patients with primary gliomas and excluded patients with metastases, arteriovenous malformations and other brain lesions from our dataset. Additionally, to account for varying the neuroplastic potential of brain regions (Ius et al., 2011; Herbet et al., 2016), we assessed the tumor extent of each lobe and performed the whole-brain analysis in the largest, most homogeneous subgroup only, to allow for adequate statistical power.

Given the relatively small magnitude in the shift in our findings, we considered whether the observed movement is a result of intersession variability. The adaptive beamforming approach for the location of the motor cortical network used in this study has a high reliability compared to the “gold standard” in neurosurgery—intraoperative mapping (Nagarajan et al., 2008). Moreover, the relocation of coordinates did not occur equally in all axes, but rather was limited to the left-right axis, with relatively high ICC in the other two axes. A similar finding was described in studies of patients recovering from a stroke and with low-grade gliomas (Liepert et al., 1998; Rossini et al., 1998; Duffau et al., 2002). Two studies also described the relocation of motor areas due to tumor along the inferior-superior (Wunderlich et al., 1998) and anterior-posterior (Conway et al., 2017) axes. Compared to longitudinal studies in healthy individuals, the shifts we observed were larger in magnitude, underlying their significance (Castillo et al., 2004; Schaefer et al., 2004), and making it unlikely that these shifts were a result of intersession variability.

### Clinical Implications of Hemisphere Laterality

Shifts of the primary MC were rather subtle, yet they were visible consistently in both analyses (whole brain activation maps and shifts in ipsilesional motor activation peaks). To ensure sufficient patient counts in the subgroup analysis, we restricted the whole-brain analysis of motor activation to patients with frontal-temporal-insular tumors—although not directly in the MC, insular tumors are near the corticospinal tract and hence, assessment of motor function in these tumors is crucial (Duffau et al., 2003). Besides, according to recent analyses in patients with slow-growing low-grade gliomas, a lower plastic reorganization potential was observed for subcortical tracts, as well as for primary areas, such as the precentral gyrus, making these comparable (Ius et al., 2011; Herbet et al., 2016).

When investigating the reasons for these shifts, we were able to identify several factors. The strongest effect was carried by tumor location, with frontal-parietal tumors showing larger shifts than predominantly frontal-insular tumors. We postulate that this is because motor activation peaks assess the shifts within the precentral gyrus, that are visible despite the limited reorganization potential of the primary cortex explained above, as others have suggested previously (Seitz et al., 1995; Bulubas et al., 2018).

Furthermore, the time between scans that were directly correlated to motor activation peak shifts, showing that the longer the period, the larger the ipsilesional MC shift. Here, support can be found through literature as well, stating that cortical reorganization is time-dependent, and thus occurs most dramatically in patients with slow-growing lesions (Duffau, 2001; Robles et al., 2008). This finding is of high clinical relevance, as an advanced cortical reorganization enables advanced functional compensation, leading to lower-rated functional deficits in patients with slow-growing lesions when compared to acute injuries (Desmurget et al., 2007). It is notable, though, that the magnitude of shift did not further increase with another observational period and a third surgical intervention, although conclusions are limited as this was true only for 11 patients.

Finally, in patients with motor deficits, we observed larger ipsilesional activation peak shifts than in patients with no motor impairment. Accordingly, in patients recovering from post-stroke motor deficits, studies reported enlarged motor areas in lesioned hemispheres (Weiller et al., 1993; Traversa et al., 1997; Liepert et al., 1998). Other MEG studies have reported that changes in β-power were observed in affected hemispheres of stroke patients, which correlated with motor function impairment (Tecchio et al., 2006, 2007; Shiner et al., 2015). These findings again suggest that, in patients with an impairment of motor pathways severe enough to cause a clinical deficit, cortical reorganization is more likely to take place. We interpret this result as further proof that reorganizational “pressure” in the form of clinical deficit can induce motor activation shifts, and that we can observe these functional reorganizations with MEG.

The whole-brain activation maps detected motor activations in the contralateral MC (ipsilateral to active finger) as well. This has been already described in healthy individuals and patients with various intracerebral lesions during a motor task (Taniguchi et al., 2000, 2004; Nagarajan et al., 2008; Willemse et al., 2010, 2016). Since ipsilateral MC activation has been demonstrated in healthy participants, it likely occurs with some frequency in the normal population. However, ipsilateral MC activation was increased in patients with structural abnormalities, compared to those with non-lesional neurologic disorders (Willemse et al., 2016). In our cohort, this observation was even stronger in the ipsilateral, and simultaneously contralesional (i.e., healthy) hemisphere. This increased ipsilateral activation might be understood as a “reservoir of functional reserve”. How frequently this phenomenon occurs is unclear; one early study found such activations in the affected side in each of six patients suffering from peri-Rolandic glioma (Taniguchi et al., 2004). Subsequent studies, however, have reported significantly lower rates of ipsilateral motor cortical network activation in the perilesional cortex (Nagarajan et al., 2008; Willemse et al., 2016).

In the group analysis of motor maps of primary MC tumors, we saw decreased activation predominantly in the healthy ipsilateral motor cortical network (Figure 3). To a lesser extent, the ipsilateral disengagement was visible when it was also the ipsilesional hemisphere, in particular in left-sided tumors (Figure 1A, “Left-sided tumors,” left hemisphere). Several studies in patients with brain tumors have shown that both hemispheres can compensate for impaired function in the primary MC (Weiller et al., 1993; Seitz et al., 1995; Duffau et al., 2002; Bulubas et al., 2016). It seems, therefore, that the intuitive reorganizational process (recruitment of contralesional motor pathways to compensate for diseased ipsilesional motor pathways) accounts for only part of the adaptation. Rather, it may be that the presence of the brain tumor itself (as well as, perhaps, the surgical resection and subsequent adjuvant therapies) induces changes in both hemispheres. Indeed, the non-primary hemisphere seems to be a reservoir of functional reserve into which the motor system can tap when it begins to experience reorganizational “pressure.”

Mechanisms aside, it is clear that a patient’s capacity for functional reorganization has a profound impact on the morbidity of his or her disease. Understanding the importance of time as factors that influence plasticity allowed in the past the introduction of multi-staged surgical resections, when, at the time point of the first surgery, no total resection is possible due to impairment of eloquent areas (Duffau et al., 2002; Robles et al., 2008). A MEG-based approach such as the one described in this article can play an important role in tracking the progress of functional reorganization in a clinical, naturalistic sample.

### Sources of Variability That Contribute to Brain Reorganization

A wide variety of sources of variability may impact brain reorganization observed in this study. We attempted to limit the interindividual variety by including only patients with primary brain tumors and considering the precise location of the tumor in the brain. One potential concern is our inability to perform the whole brain activation analysis in other subgroups than the frontal-temporal-insular tumors, as opposed to tumors directly located in the MC; yet also the non-perirolandic tumors might influence the motor pathways due to the proximity to the corticospinal tract. For spatial activation analysis, larger numbers of patients are needed to maintain power. Hence, these results need to be validated in further studies. A factor that remains is variability in the timing between the first and second (or third) MEG scan. While reorganization may be a gradual linear process, it may also mainly occur during the first weeks following surgery, or may evolve in a completely different way; the wide range of time windows here makes determining the precise temporal evolution of the functional reorganization impossible and should be investigated by future studies with controlled, repeated MEG measurements. For clinical factors, tumor location in the right and left, or motor-dominant and non-dominant hemisphere, could also introduce variability (Volkmann et al., 1998; Jung et al., 2003; Pool et al., 2014), although no influence was observed here and in our previous studies (Nagarajan et al., 2008). Furthermore, aging is associated with changes in motor beta ERD, as well as a decrease in motor cortical plasticity, and might be a source of reorganization itself (Rossiter et al., 2014; Mary et al., 2017; Rueda-Delgado et al., 2019). It is also possible that the biological manifestation of motor cortical network reorganization in our cohorts could arise from a signal to noise difference in network activation, perhaps superimposed upon the effects observed here. The effects of radiotherapy and chemotherapy on brain plasticity are unknown; although cognitive impairment hereafter can be discussed as a sign of reduced plasticity (Barz et al., 2018). Radiotherapy might also increase the risk of edema and local necrosis, causing further shifts in the MC (Hou et al., 2006). Moreover, the glioma entity influences the effects of a tumor on surrounding brain tissues and also the time a lesion needs to develop, hence possibly influencing the functional reorganization (Duffau, 2006).

Additionally, it would be of interest to include intraoperative findings into our analysis, such as the tumor resection volume, and to include a comparison of intraoperative DCS motor maps from the same time points. Unfortunately, we were not able to include this information in this study. The impact of intraoperative findings should be addressed in further studies.

## Data Availability Statement

The datasets generated for this study are available on request to the corresponding author.

## Ethics Statement

The studies involving human participants were reviewed and approved by Institutional Review Board at UCSF. The patients/participants provided their written informed consent to participate in this study.

## Author Contributions

SK, MB, SN, and PT contributed to the conception and design of the study. LB, NS, TT, AF, DM, and SH organized the database. LB and NS performed the statistical analysis. LB wrote the first draft of the manuscript. LB, NS, AF, SN, and PT wrote sections of the manuscript. All authors contributed to manuscript revision, read and approved the submitted version.

## Conflict of Interest

The authors declare that the research was conducted in the absence of any commercial or financial relationships that could be construed as a potential conflict of interest.

## Footnotes

^1^http://nutmeg.berkeley.edu

^2^http://www.fil.ion.ucl.ac.uk/spm

^3^http://www.fil.ion.ucl.ac.uk/spm

^4^http://nutmeg.berkeley.edu

## Abbreviations

ANOVA: one-way analysis of variance
BA: Brodmann area
BMRC: British Medical Research Council
MC: Motor cortex
DCS: Direct cortical stimulation
EEG: Electroencephalography
EMG: Electromyography
ERD: Event-related desynchronization
ERS: Event-related synchronization
ICC: Interclass correlation coefficient
MEG: Magnetoencephalography
MRI: Magnetic resonance imaging
nTMS: Navigated transcranial magnetic stimulation
SAM: Synthetic aperture magnetometry.
