# Supplementary material for: Motor Cortical Network Plasticity in Patients With Recurrent Brain Tumors
Source: Front Hum Neurosci. 2020 Apr 3;14:118. doi: 10.3389/fnhum.2020.00118 (PMC7146050; doi:10.3389/fnhum.2020.00118)
Supplement: Supplementary file 1 [file Table_1.DOCX]

Motor cortical network plasticity in patients with recurrent brain tumors

**Supplemental information**

1) Study flowchart with eligible subjects per analysis

Originally listed subjects (n=99)

Eligible for further analysis (n=86)

Analysis

“Activation peak analysis” (n=75)

- Two timepoints available (n=75)

- Three timepoints available (n=11)

Excluded (n=13)

- Low quality or no motor MEG recordings (n=10)

- Bilateral tumor (n=3)

Excluded (n=51)

- Tumor location group with n<20

- frontal (n=19)

- frontal-parietal (n=6)

- parietal (n=8)

- temporal (n=7)

- insular (n=7)

- other (n=12)

“Whole brain analysis” (n=27)

- Frontal-insular- temporal tumors (n=27)

Excluded (n=11)

- Inability to localize motor cortex peak activation

Intersection (n=78)

2) Peak regions with significant power decreases (refers to Figure 1)

3) Peak regions with significant power decreases (refers to Figure 3)
